# Lyme Carditis With Complete Heart Block

**DOI:** 10.7759/cureus.80724

**Published:** 2025-03-17

**Authors:** Ashot Batikyan, Hakob Harutyunyan, Vahagn Tamazyan, Aleksan Khachatryan, Pavel Abalyan, Pawel Borkowski

**Affiliations:** 1 Department of Internal Medicine, Albert Einstein College of Medicine, Jacobi Medical Center/North Central Bronx Hospital, Bronx, USA; 2 Department of Internal Medicine, Maimonides Medical Center, Brooklyn, USA; 3 Department of Cardiovascular Medicine, Icahn School of Medicine at Mount Sinai/Mount Sinai Hospital, New York City, USA; 4 Department of Medicine, Vanadzor Medical Center, Vanadzor, ARM

**Keywords:** borrelia burgdorferi infection, complete atrioventricular block, lyme carditis, lyme's disease, third degree atrioventricular block

## Abstract

Lyme disease, the most common tick-borne infection in North America, can lead to multi-organ involvement, including Lyme carditis. This report describes the case of a 42-year-old male who presented with a third-degree atrioventricular (AV) block due to Lyme carditis. The patient reported a history of a recent tick bite and erythema migrans rash, followed by progressive fatigue, palpitations, and dyspnea. Initial electrocardiogram (EKG) revealed third-degree AV block with ventricular escape rhythm, necessitating temporary transvenous pacing and intravenous ceftriaxone therapy. Within 48 hours, the patient significantly improved, transitioning to first-degree AV block with a decreasing PR interval. After clinical stabilization, intravenous ceftriaxone was switched to oral doxycycline, and the patient was discharged with outpatient follow-up. This case emphasizes the significance of early recognition and treatment of Lyme carditis to prevent life-threatening complications and avoid unnecessary permanent pacemaker implantation.

## Introduction

Lyme disease is the most common tick-borne infection in North America [1]. In 2022, over 63,000 cases were reported by the Centers for Disease Control and Prevention by state health departments and the District of Columbia [2]. It is caused by *Borrelia burgdorferi*, which is transmitted through the bite of the *Ixodes scapularis* and *Ixodes pacificus* ticks [3]. Lyme disease undergoes three distinct stages, each affecting multiple organ systems at different points in time. Weeks to months following the initial tick bite, spirochetes can disseminate to multiple organs, including the heart, joints, and nervous system [4]. The most common manifestation of Lyme carditis is atrioventricular (AV) block (90%) with high-grade AV block being responsible for more than 2/3 of cases [5,6].

Despite its rarity, Lyme carditis poses a significant diagnostic challenge due to its clinical presentation, which frequently overlaps with other conditions. Symptoms such as fatigue, palpitations, and syncope may be nonspecific, and classic signs of Lyme disease, like the erythema migrans rash, may not always be evident. Moreover, the absence of definitive diagnostic guidelines further complicates the timely recognition of this condition. However, tools like the Suspicious Index in Lyme Carditis (SILC) score and confirmatory Lyme serology can assist in diagnosis [7]. Early initiation of appropriate antimicrobial therapy and temporary pacing can effectively resolve conduction abnormalities, eliminating the need for permanent pacemakers [6]. This report presents a case of Lyme carditis presenting with third-degree AV block, providing a comprehensive review of the diagnostic approach, management, and prognosis based on relevant literature.

## Case presentation

A 42-year-old male patient with a past medical history of anxiety disorder presented to the emergency department after experiencing worsening shortness of breath on minimal exertion. He reported a possible tick bite and erythematous “bull's-eye” rash on his back two to three weeks before the presentation while visiting Long Island, New York. Since then, he has had progressive fatigue and generalized weakness. A few days ago, he started to experience palpitations, presyncope, and worsening shortness of breath with minimal exertion. Denies any chest pain, joint pain, fever, or chills.

**Figure 1:**
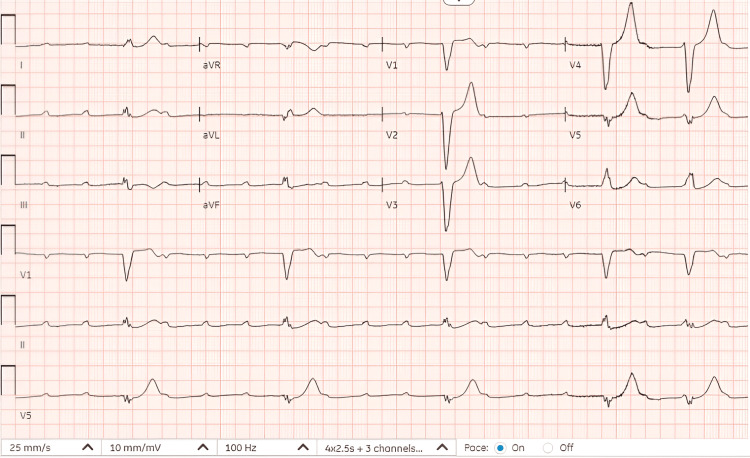
Third-degree atrioventricular block with ventricular escape rhythm; heart rate of 26 beats per minute.

Upon presentation to the emergency department, vitals were notable, with a heart rate of 57, blood pressure of 110/52, respiratory rate of 16, oxygen saturation of 100%, and temperature of 97.8°F. Physical examination revealed irregular rhythm, bradycardia, and an erythematous, circular, blanching target-like lesion with central clearing in the left thoracolumbar area. There was no jugular venous distention, heart murmurs, gallop, rub, or pedal edema. EKG upon presentation is shown in Figure 1.

On presentation, troponin was undetectable. Other remarkable laboratory tests on presentation are depicted in Table 1. Chest X-ray revealed mild pulmonary vascular congestion.

**Table 1:** Abnormal laboratory results on presentation BUN: blood urea nitrogen; hsCRP: high-sensitivity C-reactive protein; proBNP: pro-B-type natriuretic peptide

Laboratory parameter	Abnormal result	Reference range
ProBNP	2405 pg/mL	1-125 pg/mL
Creatinine	1.4 mg/dL	0.7-1.2 mg/dL
BUN	27 mg/dL	5-26 mg/dL
hsCRP	39 mg/L	<1 mg/L

**Figure 2:**
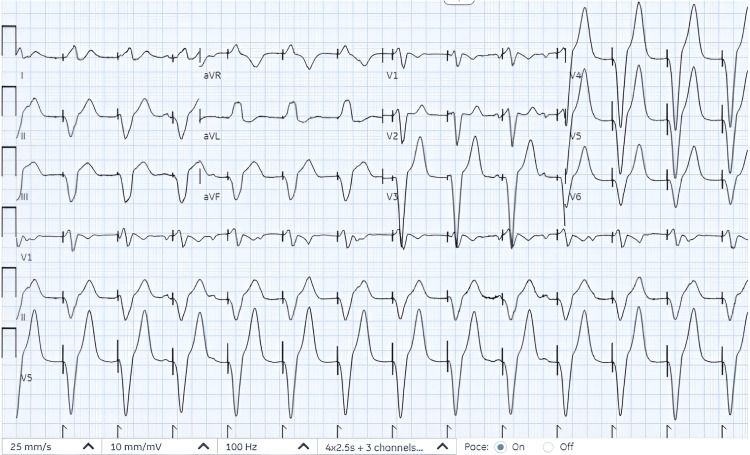
Paced rhythm with heart rate of 80 beats per minute.

While placing transcutaneous pacemaker pads, the patient vomited and had a brief cardiac arrest with seizure-like activity for 20 seconds. His blood pressure was improved with pacing and intravenous fluids. The patient was transferred to the critical care unit for the placement of an emergent transvenous pacemaker which was performed without complications. Initially, the heart rate was set for 80 beats per minute and later adjusted (Figure 2).

The patient was started on ceftriaxone 2 g intravenously daily. Lyme IgG and IgM enzyme-linked immunosorbent assay (ELISA) and Western blot tests, performed according to the CDC two-tier testing protocol for Lyme disease, were positive, confirming exposure to *Borrelia burgdorferi*. Transthoracic echocardiography was unremarkable.

**Figure 3:**
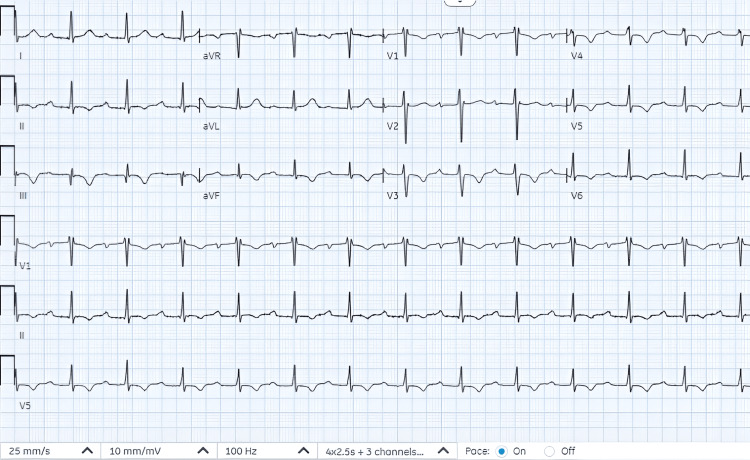
EKG at 48 hours postadmission showed narrowed complex sinus rhythm with first-degree heart block (PR interval 330 milliseconds) with diffuse ST-T wave changes.

A review of the telemetry recordings at 24 hours showed predominantly native narrow complex QRS. By 48 hours postadmission, there was no evidence of any pacemaker activity (Figure 3).

On the fourth day of hospitalization, the PR interval had improved to 290 milliseconds with narrow complex regular rhythm. Intravenous ceftriaxone, administered for five days, was switched to oral doxycycline as soon as high-grade AV block resolved and the PR interval decreased below 300 milliseconds. The patient was discharged on oral doxycycline 100 mg twice daily for two weeks, with outpatient follow-up.

## Discussion

Epidemiology

Lyme disease is a tick-borne infection caused by *Borrelia burgdorferi*, which is transmitted through the bite of the *Ixodes scapularis* and *Ixodes pacificus* ticks [3]. It is the most commonly reported vector-borne disease in North America [1]. Over 63,000 cases of Lyme disease (incidence = 18.8 cases per 100,000 population) were reported to the Centers for Disease Control and Prevention (CDC) by state health departments and the District of Columbia in 2022. Recent estimates employing alternative methodologies indicate that approximately 476,000 individuals in the United States are diagnosed and treated for Lyme disease annually. Lyme carditis, a complication of Lyme disease, is reported in approximately one out of every hundred cases reported to CDC [2] although some studies estimated the prevalence of asymptomatic carditis as high as 30% [8]. Lyme carditis is much less prevalent in Europe than in the United States, which may be explained by differences in strains of *Borrelia burgdorferi* found in Europe and North America [9].

It is more common among males between 20 and 39 years, females between 25 and 29 years, and people aged ≥75 years [10]. The reported male-to-female ratio is 2.3:1 [11]. Lyme carditis is most commonly presented by AV block (90%), with high-degree AV block accounting for approximately 2/3 of cases [5,6]. In Forrester and Mead’s retrospective review of 45 Lyme carditis cases with third-degree AV block, the male-female ratio was 5.4:1, and the most commonly involved age group was 30-39 [12].

Pathogenesis

The pathophysiology of Lyme carditis involves the colonization of cardiac tissue by *Borrelia burgdorferi*, followed by an exaggerated immune response leading to cardiac injury.

*Borrelia burgdorferi* colonization of cardiac tissue involves surface protein modulation, particularly P66, which facilitates bacterial spread and cardiac tropism. Caine and Coburn demonstrated that a strain of *Borrelia burgdorferi* lacking the expression of P66 exhibits a reduced capacity to colonize the heart in an in vivo murine model [13]. Interaction with decorin, a proteoglycan found in the extracellular matrix, is crucial for *Borrelia burgdorferi*’s heart colonization. It may also provide protection against both innate and adaptive immune responses [14-16].

Once the myocardium is colonized by *Borrelia burgdorferi*, an exaggerated immune response, characterized by activation of both innate and adaptive pathways and significant cytokine release, is triggered, leading to cardiac injury. The predominance of lymphocytes compared to sporadically identified spirochetes in Lyme carditis supports the importance of the exaggerated immune response [17,18]. Cross-reactive antibodies formed after initial exposure to bacteria may mistakenly target cardiac tissue antigens due to molecular mimicry resulting in autoimmune injury [19].

Cross-reactive antibodies formed after initial exposure to bacterial antigens may mistakenly recognize cardiac tissue antigens due to molecular mimicry, thereby contributing to autoimmune-mediated cardiac inflammation and injury.

Clinical features

According to one of the reviews of cases involving third-degree AV block associated with Lyme carditis, common symptoms at presentation were erythema migrans rash, syncope, fever, dizziness, and dyspnea. Other symptoms included weakness, palpitations, chest pain, headache, myalgia, and arthralgia. Third-degree heart block often develops within 14 days of symptom onset, with a median time of three hours in those who develop it later [12].

Diagnosis

There are currently no definitive guidelines for diagnosing and treating Lyme carditis as most of the available data comes from case reports and a limited number of large retrospective or prospective studies. Diagnosing Lyme carditis can be challenging, especially when classic signs of Lyme disease are not obvious. It requires confirmation of the association between a patient’s historical, clinical, and laboratory data [20]. Prompt diagnosis of Lyme carditis, especially manifesting as AV block, is essential to prevent life-threatening complications and unnecessary treatments like permanent pacing [6]. To address these challenges, the SILC score (Table 2) has been proposed, which may aid in identifying Lyme carditis in patients presenting with high-grade AVB, ultimately reducing the need for unnecessary permanent pacemakers [7].

Laboratory tests are supportive but not mandatory for diagnosing Lyme disease in patients from endemic areas with potential tick exposure and who have one or more skin lesions that resemble erythema migrans [21]. The primary laboratory diagnostic method for non-erythema migrans presentations of Lyme disease is screening with enzyme-linked immunosorbent assay followed by confirmation of positive or borderline positive results with Western blot assay [22]. Lyme disease serology is limited by antigenic variability and regional strain differences [23]. In addition, false-negative results can occur in early Lyme disease due to delayed immune responses, as erythema migrans usually precedes the appearance of detectable antibodies [24]. However, for early disseminated Lyme disease including Lyme carditis the sensitivity of Lyme serology is 73-100% [25].

Lyme disease serology is limited by antigenic variability and regional strain differences. False-negative results can occur in early Lyme disease due to delayed immune responses.

In addition, 12-lead EKG should be performed only in patients with signs or symptoms consistent with Lyme carditis [21]. Other tests, such as echocardiography and chest radiography, could also be useful to assess cardiac anatomic or functional abnormalities.

Routine endomyocardial biopsy is not recommended for diagnosis of Lyme carditis given the potential focalization of myocarditis and the high risk associated with the procedure [20], although it can be considered in certain cases [26].

Treatment

The mainstay of managing Lyme carditis is supportive care and antimicrobial therapy. Symptomatic patients with Lyme carditis as well as those with high-grade AV block or first-degree AV block with PR interval more than 300 msec should be hospitalized with continuous EKG monitoring. For outpatients with Lyme carditis, oral antibiotics might be preferred. For hospitalized patients, initial treatment with intravenous ceftriaxone is recommended over oral antibiotics. After clinical improvement is achieved, the treatment can be completed with oral antibiotics. Antibiotics used for Lyme carditis are intravenous ceftriaxone and oral doxycycline, amoxicillin, cefuroxime axetil, and azithromycin [3,21].

Patients who have high-degree AV block or cardiac symptoms along with intermediate or high SILC score suggestive of Lyme disease should receive immediate empiric IV ceftriaxone while their Lyme serology is being processed. For Lyme carditis patients with symptomatic high-grade AV block or those with high-risk electrocardiographic findings such as alternating bundle branch block, temporary pacing modalities rather than implanting a permanent pacemaker should be used. After serological confirmation of Lyme disease, intravenous ceftriaxone should be continued, followed by oral antibiotics once clinical improvement is observed to complete a two to three weeks of therapy [21].

As most cases of heart block resolve within six days, a permanent pacemaker is not recommended unless 1:1 AV conduction is not restored within 14 days of admission [12]. A stress test is recommended 10 days after admission for patients with resolved heart block to assess AV conduction. If 1:1 AV conduction is maintained for more than 120 beats per minute, the patient can be discharged with an oral antibiotic.

However, if conduction fails at less than 90 beats per minute, a permanent pacemaker may be recommended. If the Wenckebach point, where the AV node starts to exhibit decremental conduction, occurs between 90 and 120 beats per minute, it is recommended to repeat the stress test in four to six weeks before considering permanent pacing. Typically, conduction improves notably beyond the Wenckebach point, making permanent pacing rarely necessary. An outpatient follow-up should be scheduled six weeks after discharge to ensure there are no persistent rhythm or conduction issues [27].

Prognosis

The prognosis of Lyme carditis in general is excellent. Although there are few case reports of patients with Lyme carditis needing permanent pacing despite correct antibiotic therapy, this is rare. In van der Linde’s retrospective review of 105 Lyme carditis cases, 94% of patients recovered completely. A permanent pacemaker was implanted in five patients, but in four patients the conduction disturbances resolved completely and one patient had persistent complete heart block [5]. In Forrester’s retrospective review of 45 Lyme carditis cases with third-degree AV block, a permanent pacemaker was inserted in only two patients [12].

## Conclusions

This case underscores the critical importance of early recognition and timely management of third-degree AV block caused by Lyme carditis to prevent unnecessary permanent pacemaker placement. In cases with high suspicion of Lyme carditis presenting with high-grade AV block, prompt initiation of empiric antibiotic therapy is recommended even before Lyme serology results are available. Temporary pacing, rather than a permanent pacemaker, should be used in patients with Lyme carditis who present with symptomatic high-grade AV block or high-risk electrocardiographic findings such as alternating bundle branch block. With the timely initiation of antibiotics, the overall prognosis for these patients is favorable, with only a small proportion requiring permanent pacemaker placement.
